# Temporal variation in out-of-hospital cardiac arrest occurrence in individuals with or without diabetes

**DOI:** 10.1016/j.resplu.2021.100167

**Published:** 2021-09-22

**Authors:** L.H. van Dongen, P. de Goede, S. Moeller, T.E. Eroglu, F. Folke, G. Gislason, M.T. Blom, P.J.M. Elders, C. Torp-Pedersen, H.L. Tan

**Affiliations:** a Amsterdam UMC, Academic Medical Center, University of Amsterdam, Department of Experimental and Clinical Cardiology, Heart Centre, Amsterdam Cardiovascular Sciences, Meibergdreef 9, Amsterdam, the Netherlands; b Laboratory of Endocrinology, Amsterdam University Medical Center, University of Amsterdam, Amsterdam Gastroenterology, Endocrinology and Metabolism, Amsterdam, the Netherlands; c Hypothalamic Integration Mechanisms Group, Netherlands Institute for Neuroscience (NIN), an Institute of the Royal Netherlands Academy of Arts and Sciences, Amsterdam, the Netherlands; d Department of Cardiology, Copenhagen University Hospital Gentofte, Hellerup, Denmark; e Emergency Medical Services Copenhagen, University of Copenhagen, Denmark; f The Danish Heart Foundation, Copenhagen, Denmark; g Amsterdam UMC, Vrije Universiteit Amsterdam, Department of Epidemiology and Biostatistics, Amsterdam Public Health Research Institute, De Boelelaan 1117, Amsterdam, Netherlands; h Amsterdam UMC, Vrije Universiteit Amsterdam, Department of General Practice Medicine, Amsterdam Public Health Institute, De Boelelaan 1117, Amsterdam, Netherlands; i Department of Cardiology, North Zealand Hospital, Hillerød, Denmark; j Department of Cardiology, Aalborg University Hospital, Aalborg, Denmark; k Department of Public Health, University of Copenhagen, Denmark; l Netherlands Heart Institute, Utrecht, the Netherlands

**Keywords:** Out-of-hospital cardiac arrest, Diabetes mellitus, Circadian rhythm, Temporal variation

## Abstract

**Objective:**

Out-of-hospital cardiac arrest (OHCA) occurrence has been shown to exhibit a circadian rhythm, following the circadian rhythm of acute myocardial infarction (AMI) occurrence. Diabetes mellitus (DM) is associated with changes in circadian rhythm. We aimed to compare the temporal variation of OHCA occurrence over the day and week between OHCA patients with DM and those without.

**Methods:**

In two population-based OHCA registries (Amsterdam Resuscitation Studies [ARREST] 2010–2016, *n* = 4163, and Danish Cardiac Arrest Registry [DANCAR], 2010–2014, *n* = 12,734), adults (≥18y) with presumed cardiac cause of OHCA and available medical history were included. Single and double cosinor analysis was performed to model circadian variation of OHCA occurrence. Stratified analysis of circadian variation was performed in patients with AMI as immediate cause of OHCA.

**Results:**

DM patients (22.8% in ARREST, 24.2% in DANCAR) were older and more frequently had cardiovascular risk factors or previous cardiovascular disease. Both cohorts showed 24 h-rhythmicity, with significant amplitudes in single and double cosinor functions (*P*-range < 0.001). In both registries, a morning peak (10:00–11:00) and an evening peak (20:00–21:00) was observed in both DM and non-DM patients. No septadian variation was observed in either DM or non-DM patients (*P*-range 0.13–84).

**Conclusions:**

In these two population-based OHCA registries, we observed a similar circadian rhythm of OHCA occurrence in DM and non-DM patients.

## Introduction

Several studies have shown circadian and septadian variation in the occurrence of out-of-hospital cardiac arrest (OHCA), with peak incidence during morning hours and on Mondays.^1^, ^2^, ^3^, ^4^ However, two recent studies have failed to observe these peaks, and observed an overall higher occurrence during daytime and a nadir on Sundays.^5^, ^6^ The circadian and septadian variations of OHCA incidence seem to coincide with the temporal variations in the incidence of acute myocardial infarction (AMI),^7^, ^8^ a major cause of OHCA. It is possible that the temporal variation in OHCA incidence is due to temporal variation in cardiac (electro)physiologic properties that are involved in the occurrence of the cardiac arrhythmias that underlie OHCA.^9^, ^10^, ^11^ However, a previous study suggested that underlying patient characteristics need to be explored to provide further insight into the observed temporal patterns.^12^

One patient characteristic that may be associated with changes in temporal variation is diabetes mellitus (DM). Patients with DM have a 2–3 fold increased risk for OHCA,^13^, ^14^, ^15^ and previous studies have shown that DM is associated with changes in the temporal patterns of various parameters that impact on the occurrence of cardiac arrhythmias, e.g.,^16^, ^17^ blood pressure,^18^, ^19^ hypoglycaemia,^20^ and QTc duration.^21^ Moreover, QTc prolongation in DM seems to be associated with cardiac autonomic neuropathy and has been demonstrated in DM patients without any evidence of ischemic heart disease.^22^ Also, DM is associated with attenuated day-night differences in the sympathovagal balance,^23^ and one study showed no circadian rhythm of AMI occurrence in DM patients without neuropathy.^24^ These disruptions and the absence of a circadian rhythm of AMI in DM might result in diminished circadian and septadian variation in OHCA occurrence or different timing of OHCA events as compared to a non-diabetic OHCA population.

With this study, we aimed to investigate the temporal variation of OHCA occurrence over the day and week by studying patients with or without DM in a Dutch and a Danish OHCA registry. Additionally, as OHCA occurrence has been observed to follow the temporal variation of AMI, AMI is a major cause of OHCA, and OHCA in patients with DM is often attributed to increased risk of AMI,^25^ we investigated whether such variations occur among OHCA patients in whom AMI was the immediate cause of OHCA.

## Methods

### Study population

The patients included in this study were drawn from the Amsterdam Resuscitation Studies (ARREST) and the Danish Cardiac Arrest Registry (DANCAR). ARREST is an ongoing prospective registry of all Emergency Medical System (EMS)-attended OHCA cases in one contiguous region of the Netherlands (North Holland province, urban and rural areas, 2.4 million inhabitants).^26^ DANCAR is a nationwide registry holding information on all OHCA patients in Denmark with a resuscitation attempt either by bystander or EMS.^27^

Patients with a presumed cardiac cause of OHCA above the age of 18 were included from the ARREST registry in 2010–2016 and from DANCAR in 2010–2014. ARREST and DANCAR were conducted according to the Declaration of Helsinki. The ARREST registry was approved by the Medical Ethics Committee of the Academic Medical Centre in Amsterdam (Ref.no.2017_260), and written informed consent was obtained from all surviving patients. Registry-based studies in Denmark do not require ethical approval, however, DANCAR was approved by the Danish Data Protection Agency (Ref.no. 2007-58-0015, local ref.no. GEH-2014-017, I-Suite.no. 02735). This study falls under the approval of the ARREST and DANCAR registries.

### Data collection

In ARREST, age and sex was retrieved from EMS and hospital information. Medical history was retrieved from the general practitioner (GP) and contained the patients’ episode list and/or letters from medical specialists, along with a questionnaire filled out by the GP. This questionnaire contained a checklist of several comorbidities, including heart diseases, cardiovascular risk factor and other comorbidities. Patients were classified as having DM if the GP stated the patients had either type 1 or type 2 DM prior to OHCA. For the Danish population we linked the cardiac arrest patients from DANCAR to information from other national registries using the unique civil registration number that is assigned to all Danish residents. By this we obtained information on age and sex from the Civil Personal Registration Registry.^28^ We obtained information on hospital admissions, including discharge diagnosis codes, which were used to define risk factors and comorbidities (all included diagnosis codes are in accordance with the International Classification of Diseases (ICD-10) system).^29^, ^30^ We obtained information about medication use up to 180 days before OHCA from the National Prescription Registry. This information was combined with discharge diagnosis codes to define history of DM and hypertension, as well as hypercholesterolemia including patients with lipid-lowering medication use.^31^, ^32^ Due to low prevalence of T1D and our assumption that both in T1D and T2D similar processes, such as diabetic neuropathy, may affect cardiac electrophysiology,^33^ T1D and T2D are combined in the DM group.

For both registries the resuscitation parameters included bystander or ambulance witnessed OHCA (yes/no), OHCA location (home vs. public), bystander cardiopulmonary resuscitation (CPR, yes/no), connection of an automated external defibrillator (AED, yes/no, in ARREST by bystander or first responder, in DANCAR by bystander when shocked) and response time (in ARREST time from emergency call to time of connection of AED or manual defibrillator, whichever came first, in DANCAR: time from emergency call to first rhythm analysis by the EMS), and were collected according to the Utstein criteria.^34^ OHCA date and time were retrieved from the EMS dispatch centre for ARREST and DANCAR. Time of OHCA was categorized per hour starting from midnight.

AMI as immediate cause of OHCA could only be established for patients who survived to hospital admission. In ARREST this was derived from hospital records (including letters, ECGs, cardiac enzymes, treatments, discharge diagnosis and death certificates). In DANCAR, this was derived from ICD10 diagnosis codes (DI21) and death certificate diagnosis.

### Statistical analysis

Descriptive statistics are summarized as means ± SD, or medians with interquartile ranges (IQR) for continuous variables. Categorical variables are presented as frequencies and percentages. Both cohorts are presented separately, due to different population characteristics and definitions.

OHCA occurrence by DM status was visualized by calculating the proportion per hour of the day, and day of the week and plotting these proportions per hour/day and fitting a single and double cosinor. Previous studies suggested a single cosinor would provide a suitable fit to analyse OHCA occurrence. Therefore, we initially analysed OHCA occurrence using a single cosinor,^8^, ^35^ fitting the data to the following regression: *y* = *a* + b*cos (2*π*(*x* − *c*)/24), where a is the mesor (mean) level, b the amplitude (difference between mean and peak), and c the acrophase (time of peak) of the rhythm.^36^, ^37^ However, our data points suggested that a double cosinor could provide a better fit, and consequently we performed a double cosinor as a second analysis, using the following regression: *y* = *a* + b*cos(2*π*(*x* − *c*)/24) + d*cos(4*π*(*x* − e)/24), where a is the mesor (mean), b the amplitude of the first cosine function, *c* the acrophase (top) of the first cosine function, d the amplitude of the second cosine function, and *e* the acrophase (top) of the second cosine function. For the septadian analysis, data were fitted to *y* = *a* + b*cos(2*π*(*x* − *c*)/7). To numerically solve for the peaks of the fitted double cosine the local maxima were determined using Python (script available upon request). These analyses were performed in several populations in both cohorts individually: the total study population, a subgroup of patients with an AMI as immediate cause of their OHCA, and in a 1:1 matched population, as DM patients had different characteristics as compared to non-DM patients. Cosinor analysis does not allow for direct adjustment for confounders. Nevertheless, in attempt to adjust the cosinor analysis for confounding, 1:1 propensity score matching has been performed to match a case to a control, with a calliper of 0.1. In the ARREST cohort, the model included age, sex, cardiovascular disease risk factors (obesity, hypertension, hypercholesterolemia, in a cumulative manner) and cardiovascular diseases (previous AMI, previous stroke, in a cumulative manner). For DANCAR we were not able to obtain data on obesity, but otherwise the model included the same variables. Lastly, a sensitivity analysis has been performed in only T2D patients, thereby excluding T1D patients, in the total population.

Statistical analyses were carried out using SPSS Statistics version 26.0 software (SPSS Inc, Chicago, IL), R version 3.4.1 (“R Development Core Team”)^38^ and the cosinor analyses with Sigmaplot 14.0 (Systat Software Inc, San Jose, CA) and Python version 3.9.2 (Python Software Foundation). A two-sided *p* < 0.05 was considered statistically significant.

## Results

In the ARREST registry, 4,163 patients were included with a presumed cardiac cause of OHCA, complete medical history present and known time of call to EMS dispatch centre (eFigure 1). Of these patients, 22.8% (*n* = 949) were diagnosed with DM. In DANCAR, 12,734 patients met the inclusion criteria, and 24.2% (*n* = 2,481) were diagnosed with DM (eFigure 2).

Overall, patients with DM were older, had more cardiovascular risk factors and more often previous cardiovascular disease (Table 1). Additionally, patients with DM were less frequently in a public location when OHCA struck, and less frequently presented with a shockable initial rhythm.

**Table 1:** Baseline characteristics of the study populations.

	Arrest		Dancar*	
	Diabetics	Non-diabetics	Diabetics	Non-diabetics
	*n* = 949	*n* = 3,214	*n* = 2,481	*n* = 10,253
Age (y), mean ± SD	71.2 ± 11.2	67.0 ± 14.3	72.0 ± 11.4	71.1 ± 14.4
Male sex	645 (68.0)	2301 (71.6)	1664 (67.1)	6801 (66.3)
Cardiovascular risk factors				
Obese	356 (37.5)	443 (13.8)	-	-
Hypertension	684 (72.1)	1,396 (43.4)	1,837 (74.0)	4,576 (44.6)
Hypercholesterolemia	459 (48.4)	870 (27.1)	1,717 (69.2)	3,108 (30.3)
Previous cardiovascular disease				
Myocardial infarction	293 (30.9)	610 (19.0)	406 (16.4)	993 (9.7)
Heart failure	286 (30.1)	490 (15.2)	793 (32.0)	1,871 (18.2)
Arrhythmias	282 (29.7)	619 (19.3)	749 (30.2)	2,204 (21.5)
Cardiomyopathy	89 (9.4)	198 (6.2)	134 (5.4)	339 (3.3)
Valve disorders	173 (18.2)	402 (12.5)	275 (11.1)	886 (8.6)
CVA/TIA	177 (18.7)	367 (11.4)	443 (17.9)	1,297 (12.6)
Resuscitation characteristics				
Witnessed status†	674 (71.7)	2,356 (73.9)	1,429 (58.0)	6,116 (60.0)
Public location	208 (21.9)	899 (28.0)	553 (22.9)	2,866 (28.8)
Bystander CPR	668 (71.1)	2,371 (74.7)	1,338 (54.2)	5,512 (54.1)
AED used‡	487 (51.3)	1,703 (53.0)	64 (2.7)	335 (3.5)
Shockable initial rhythm	349 (37.1)	1,479 (46.6)	542 (22.8)	2,673 (27.3)
Response time (min), median (IQR)	8.9 (6.6–11.5)	8.5 (6.6–10.9)	12 (8–19)	13 (8–20)

Results are presented in n (%), unless specified otherwise.

Missing values ARREST: witnessed status *n* = 36, public location *n* = 1, bystander CPR *n* = 49, shockable initial rhythm *n* = 48, and response time *n* = 383. Missing values DANCAR: witnessed status *n* = 75, location of arrest *n* = 370, bystander CPR *n* = 77, bystander AED use *n* = 786, initial recorded heart rhythm *n* = 562, and EMS response time *n* = 3,525.

* Note: in DANCAR comorbidity assessment was based on hospital information, and for DM, hypertension and hypercholesterolemia medication use up to 180 days before OHCA was included.

† Witnessed by either a bystander or ambulance personnel.

‡ AED use in ARREST by both bystanders and first responders, in DANCAR only by bystanders.

**Fig. 1:**
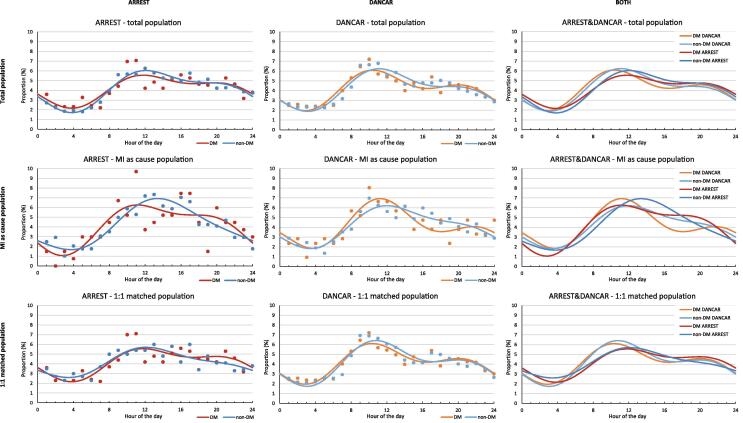
Distribution of occurrence of OHCA over the hours of the day by diabetes status using double cosinor modelling in ARREST (first column), DANCAR (second column) and a combination of both cohorts (third column) for the total population (top row), in patients with MI as cause of OHCA (middle row) and in a 1:1 matched population (bottom row). ARREST; Amsterdam Resuscitation Studies, DANCAR; Danish Cardiac Arrest Registry, DM; diabetes mellitus, MI; myocardial infarction, OHCA; out-of-hospital cardiac arrest.

Analysis of circadian variation in OHCA occurrence, fitted with a single cosinor wave, showed an amplitude statistically significantly greater than zero, indicating 24-h rhythmicity in both cohorts (*P*-range < 0.001, eFigure 3 and eTables 1–2). However, visual inspection of the data points indicated that the single cosinor fit did not capture the individual data points sufficiently well; e.g. individual data points observed in the morning and early evening were not reflected in the single cosinor fit. Accordingly, we subsequently fitted the data with a double cosinor function, and found that the individual data points were well captured using this fit (Fig. 1). We therefore used this analysis as our main analysis of circadian variation. Using this analysis, we observed a 24 h rhythmicity in both DM and non-DM patients, indicated by statistically significant amplitudes (*P*-range < 0.0004, eTable 3). Moreover, we found that, in both cohorts, the circadian variation in OHCA occurrence was similar in DM patients and in non-DM patients, with a major peak around 10:30–12:00 and a second peak around 19:00–20:00 (eTable 3). Limiting our analysis of the DM group to only T2D yielded similar results (eFigure 4). Moreover, similarly as the single cosinor analysis, the amplitude in non-DM is slightly higher (in ARREST 1.36 ± 0.25 vs. 1.79 ± 0.11 and in DANCAR 1.40 ± 0.17 vs. 1.68 ± 0.002, for DM vs non-DM, eTable 3). In the AMI population, the first peak of the DM groups are a little earlier as compared to the non-DM groups of both cohorts; in DANCAR, the DM group with AMI as cause had the peak at 11:00, and the non-DM at 12:00, and in ARREST the DM groups peaked at 11:00 and non-DM at 13:00. No second peaks could be determined for these groups (eTable 3), as after the first peak the occurrence drops and does not rise again (no local maximum), despite having a clearly visible shoulder in the curve. This resulted in the inability to solve for a second peak. In the 1:1 matched population the first peak in DANCAR lies around 10:00–11:00 am, and in ARREST around 12:00.

**Fig. 2:**
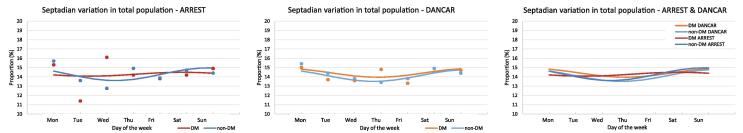
Distribution of occurrence of out-of-hospital cardiac arrest over the week by diabetes status in both cohorts using single cosinor modelling in the total population. DM; diabetes mellitus.

Analysis of septadian variation using single cosinor showed non-significant amplitudes in both DM and non-DM in the total population (Fig. 2), in both cohorts, indicating no septadian variation (eTables 1–2).

## Discussion

In these Dutch and Danish population-based cohorts of EMS-attended OHCAs, we observed a circadian rhythm of OHCA occurrence in DM and non-DM patients, with a first peak in the late morning and a second peak in the early evening. However, the circadian rhythms of patients with and without DM were comparable. No septadian variation was observed in both DM and non-DM.

### Circadian variability in OHCA occurrence

Over the past decades, several studies have demonstrated circadian patterns in the incidence of OHCA, with peaks typically in the morning hours and a smaller peak in the late afternoon/early evening.^1^, ^2^, ^3^, ^4^, ^5^ However, recently two studies showed no morning peak, but a constantly higher frequency during the wake hours.^4^, ^5^ The current study shows a 24-h rhythmicity, indicated by significant amplitudes in the cosinor analyses. Moreover, our study showed a morning peak and a smaller peak in early evening. Nevertheless, our study does concur with the abovementioned studies regarding a higher occurrence of OHCA during wake-hours.

OHCA occurrence has been known to follow the temporal variation of AMI, with peaks around 11:00.^8^, ^39^, ^40^ However, two recent studies observed something different: one study showed not only a morning peak, but an additional peak in the late afternoon,^41^ and the other showed no peak in the morning, but overall highest rates during daytime.^1^ Circadian variation in our subpopulation of patients with MI as immediate cause of OHCA, which looked very similar to the total OHCA population, showed a peak of around 10–11 in the morning, and a later peak in the late afternoon/early evening, but overall higher daytime occurrence.

Two studies investigating circadian rhythms in OHCA occurrence^2^ and ST-elevation AMI (STEMI) occurrence,^42^ including DM, showed the occurrence of both OHCA and STEMI was fairly constant over the day in DM patients. This has been attributed to defective cardiac innervation in DM or more vulnerable plaques in untreated diabetic patients, so smaller perturbations could lead to AMI.^43^ Nevertheless, in contrast with these studies, the present study did show a circadian rhythm in DM patients, which was similar to the non-DM population. This difference might be attributed the fact that the first study^2^ only looked at three time categories (daytime, evening and night), which might have resulted in a loss of temporal resolution, and the second study^42^ looked at STEMI instead of OHCA, and used frequencies instead of proportions, which might have complicated to visually compare groups. Nevertheless, in our study circadian rhythms of OHCA occurrences in DM and non-DM patients are comparable, suggesting that the changes in temporal patterns of (electro)physiologic parameters in DM do not influence the circadian rhythm of OHCA occurrence.

### Septadian variability in OHCA occurrence

Several (older) community-based studies have consistently shown a Monday peak in OHCA occurrence. This peak has been attributed to the stress of the new workweek. Nevertheless, several recent studies, did not observe a Monday peak of OHCA incidence.^1^, ^4^, ^5^ These studies reported peaks on Saturday,^1^ or in the weekend,^4^ or a nadir on Sunday.^5^ A peak in the weekend or Saturday could be explained by unhealthy habits in the weekend (e.g. alcohol consumption). However, our study did not suggest a clear septadian rhythmicity.

### Limitations

Several issues are important to note in the interpretation of the present study. First, only patients in which the EMS (or bystanders in DANCAR) have attended the OHCA are included in this study, so it may exclude or misclassify patients who died at night, but were only detected in the morning. Nevertheless, results from Jallow et al.^1^ who included also already deceased patients (with thus less certainty on the actual time of collapse), showed no tendency of more OHCAs occurring at night. Second, we could not account for severity of DM, and therefore lacked the ability to study possible changes in diurnal (or septadian) patterns of OHCA occurrence in patients with severe DM. Additionally, we have presumed comparability in temporal variation in patients with type 1 and type 2 diabetes. As T2D are the main group (97%) results are only generalizable to T2D. Third, in our analysis of patients with an AMI as immediate cause of the OHCA, only patients who have survived to hospital admission are included, as diagnosis occurs in hospital. At last, the study was based on observational data and the results therefore represent associations and not necessarily causal relations.

## Conclusion

In these two population-based OHCA registries, we observed a comparable circadian rhythm of OHCA occurrence in DM and non-DM patients with a peak in the late morning and in the early evening.

## Data availability statement

The data underlying this article are available in the article and in its online supplementary material. The data cannot be shared publicly for privacy of individuals that participated in the study as data cannot be provided completely anonymous according to the Medical Ethics Committee and the Data Protection Officer of our institution (MEC: mecamc@amc.nl, DPO: fg@amc.nl).

## CRediT authorship contribution statement

**L.H. van Dongen:** Conceptualization, Methodology, Formal analysis, Investigation, Visualization, Writing – original draft, Writing – review & editing. **P. de Goede:** Conceptualization, Methodology, Formal analysis, Software, Writing – review & editing. **S. Moeller:** Formal analysis, Investigation, Writing – original draft, Writing – review & editing. **T.E. Eroglu:** Writing – review & editing. **F. Folke:** Resources, Writing – review & editing. **G. Gislason:** Resources, Writing – review & editing. **M.T. Blom:** Conceptualization, Methodology, Writing – original draft, Writing – review & editing, Supervision. **P.J.M. Elders:** Conceptualization, Methodology, Writing – review & editing. **Christian Torp-Pedersen:** Resources, Writing – review & editing. **H.L. Tan:** Conceptualization, Methodology, Supervision, Resources, Writing – review & editing.
