# Supplementary material for: Temporal variation in out-of-hospital cardiac arrest occurrence in individuals with or without diabetes
Source: Resusc Plus. 2021 Sep 22;8:100167. doi: 10.1016/j.resplu.2021.100167 (PMC8473536; doi:10.1016/j.resplu.2021.100167)
Supplement: Supplementary data 1 [file mmc1.pdf]

eFigure 1. Flow chart of patient inclusion in ARREST.

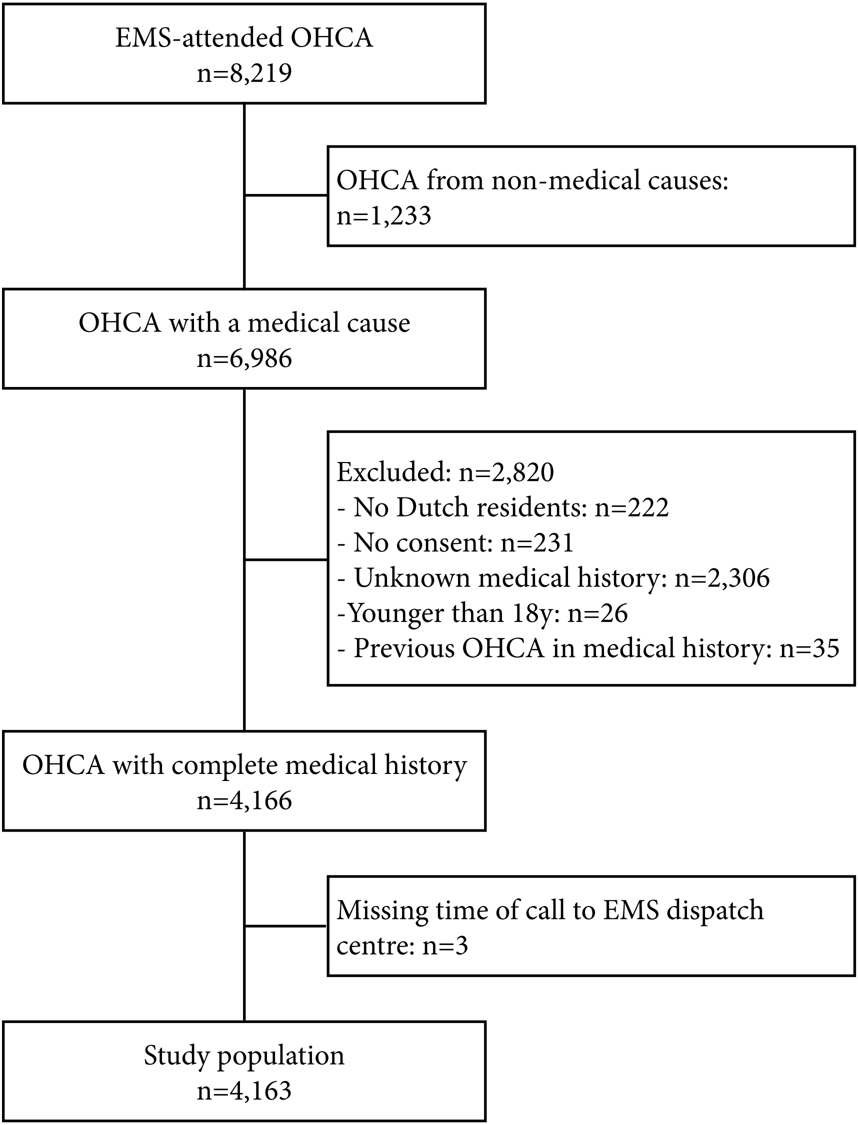

EMS = emergency medical services; OHCA = out-of-hospital cardiac arrest.
