# Supplementary figures and images for: Temporal variation in out-of-hospital cardiac arrest occurrence in individuals with or without diabetes
Source: Resusc Plus. 2021 Sep 22;8:100167. doi: 10.1016/j.resplu.2021.100167 (PMC8473536; doi:10.1016/j.resplu.2021.100167)

eFigure 2. Flow chart of patient inclusion in DANCAR.

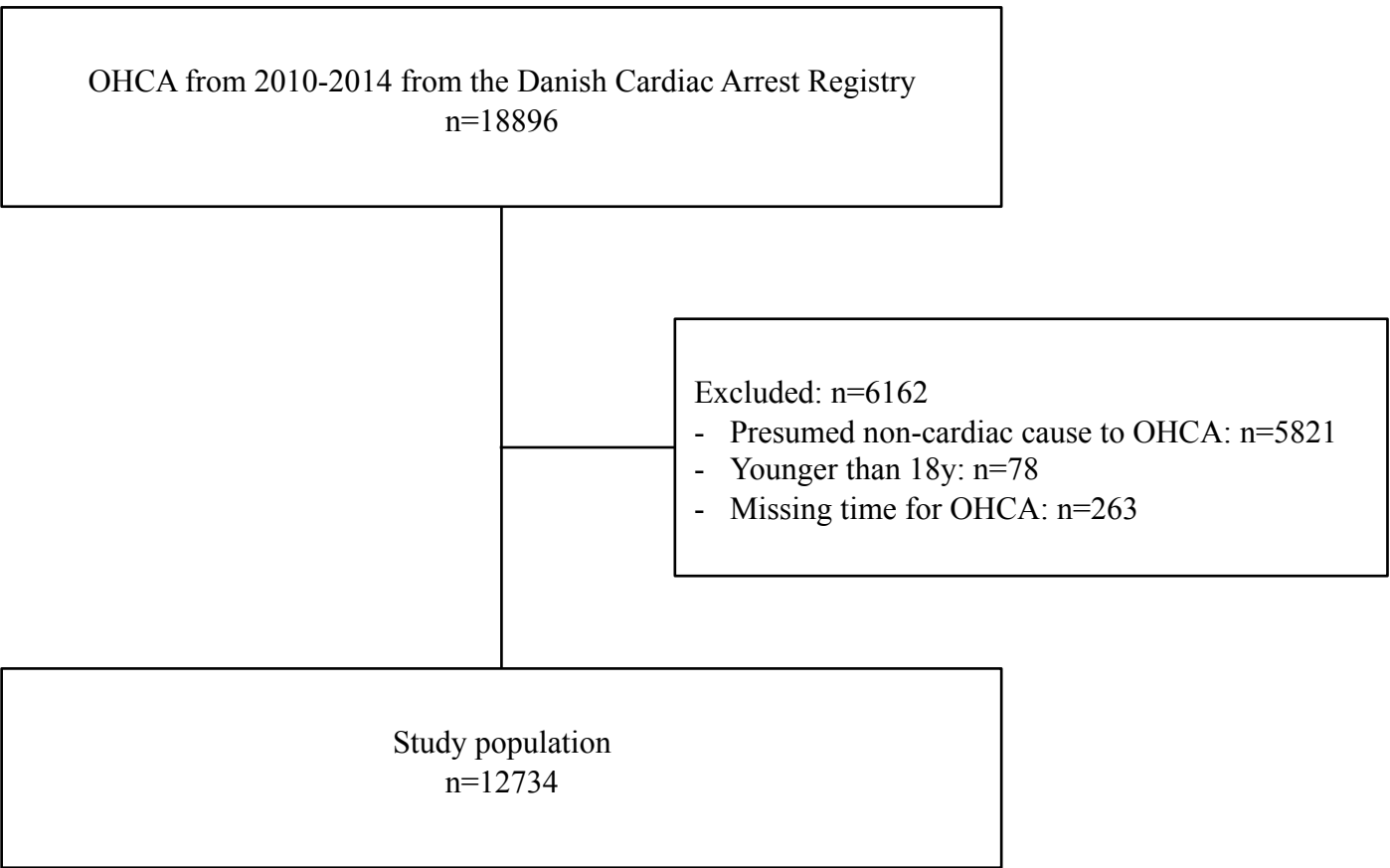

OHCA = out-of-hospital cardiac arrest

Supplement: Supplementary data 2 [file mmc2.pdf]
