# Supplementary material for: Temporal variation in out-of-hospital cardiac arrest occurrence in individuals with or without diabetes
Source: Resusc Plus. 2021 Sep 22;8:100167. doi: 10.1016/j.resplu.2021.100167 (PMC8473536; doi:10.1016/j.resplu.2021.100167)
Supplement: Supplementary data 3 [file mmc3.pdf]

eFigure 3. Distribution of occurrence of OHCA over the hours of the day by diabetes status using single cosinor modelling in ARREST (first column), DANCAR (second column) and a combination of both cohorts (third column) for the total population (top row), in patients with MI as cause of OHCA (middle row) and in a 1:1 matched population (bottom row).

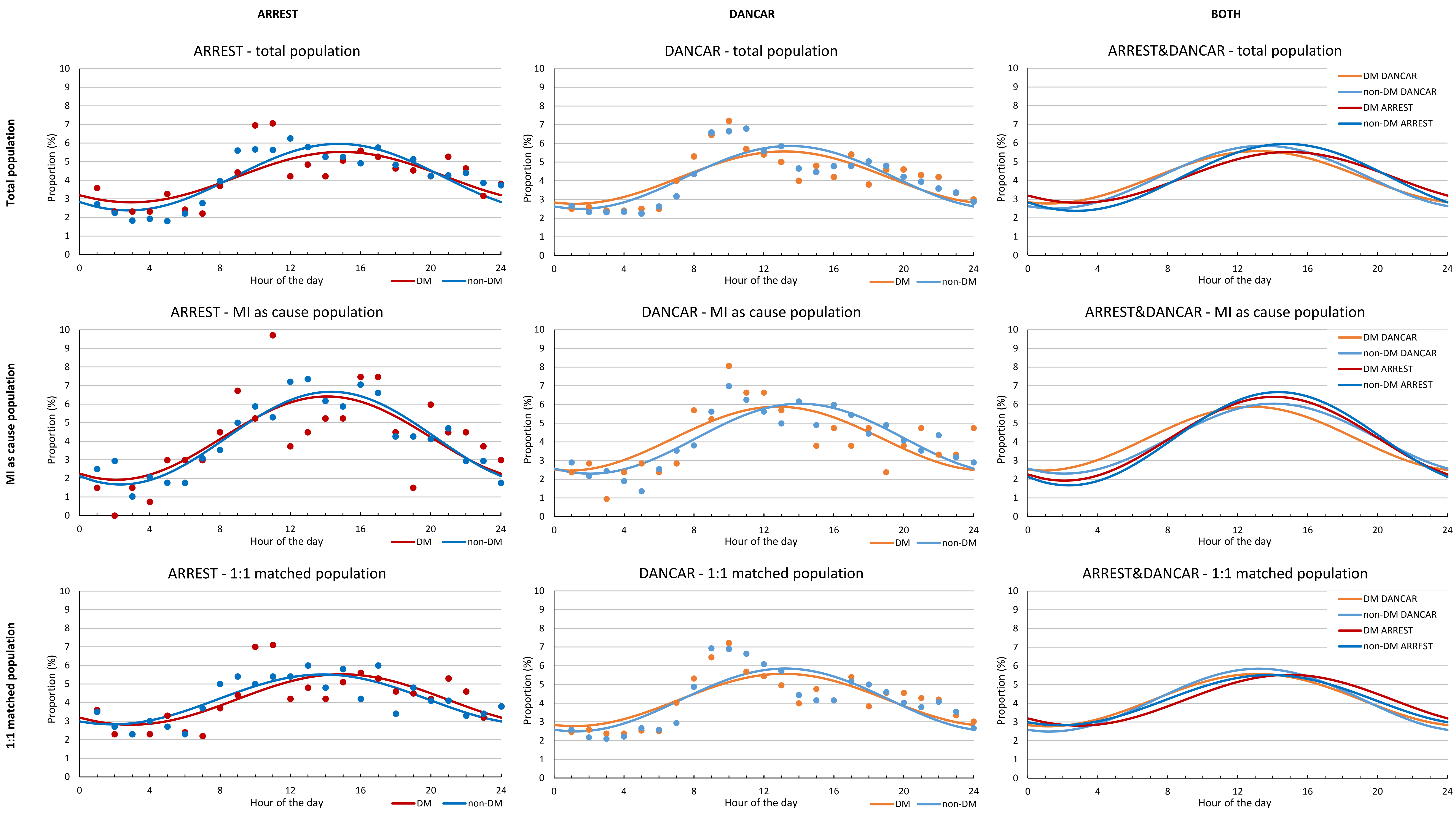

Abbreviations

ARREST; Amsterdam Resuscitation Studies, DANCAR; Danish Cardiac Arrest Registry, DM; diabetes mellitus, MI; myocardial infarction, OHCA; out-of-hospital cardiac arrest.
