# Supplementary material for: Temporal variation in out-of-hospital cardiac arrest occurrence in individuals with or without diabetes
Source: Resusc Plus. 2021 Sep 22;8:100167. doi: 10.1016/j.resplu.2021.100167 (PMC8473536; doi:10.1016/j.resplu.2021.100167)
Supplement: Supplementary data 4 [file mmc4.pdf]

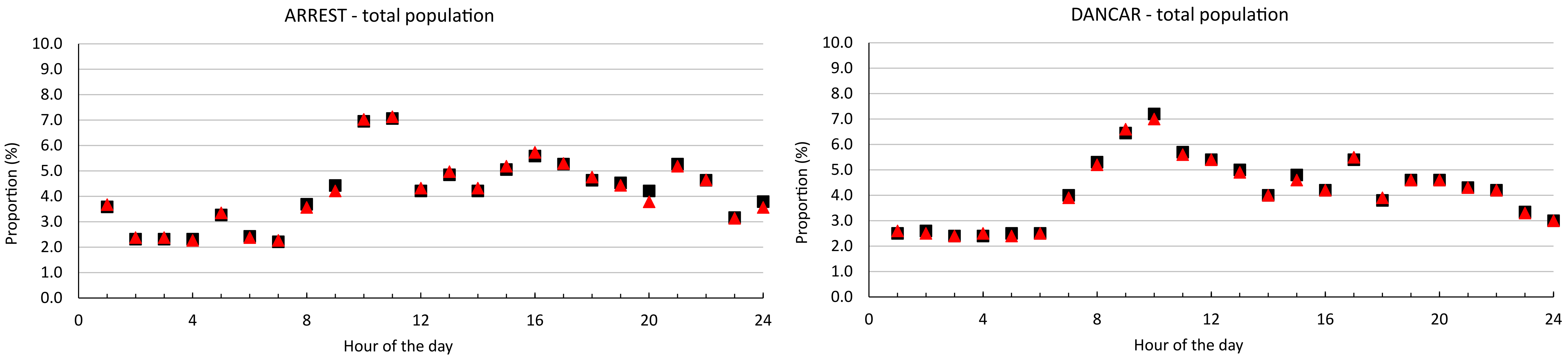

eFigure 4. Proportion of OHCA occurrence in all diabetes patients (black) and onl type 2 diabetes patients (red) in ARREST and DANCAR.
