# Supplementary material for: Temporal variation in out-of-hospital cardiac arrest occurrence in individuals with or without diabetes
Source: Resusc Plus. 2021 Sep 22;8:100167. doi: 10.1016/j.resplu.2021.100167 (PMC8473536; doi:10.1016/j.resplu.2021.100167)
Supplement: Supplementary data 5 [file mmc5.docx]

**eTable 1. Cosinor values of the OHCA occurrence shown by diabetes mellitus status in ARREST.**

| Population | Time | Cosinor parameters | Diabetics | | | Non-Diabetics | | |
| --- | --- | --- | --- | --- | --- | --- | --- | --- |
|  |  |  | Coefficient ± SE | P-value | Coefficient ± SE | | P-value |  |
| Total  (n=4,163) | Circadian | Mesor | 4.17 ± 0.20 | <0.0001 | 4.17 ± 0.14 | | <0.0001 |  |
|  |  | Amplitude | 1.36 ± 0.28 | <0.0001 | 1.79 ± 0.20 | | <0.0001 |  |
|  |  | Acrophase | 14.94 ± 0.80 | 0.0014 | 14.77 ± 0.42 | | <0.0001 |  |
|  | Septadian | Mesor | 14.29 ± 0.69 | <0.0001 | 14.29 ± 0.38 | | <0.0001 |  |
|  |  | Amplitude | 0.21 ± 0.98 | 0.84 | 0.68 ± 0.54 | | 0.27 |  |
|  |  | Acrophase | 5.84 ± 5.20 | 0.32 | 6.81 ± 0.87 | | 0.0015 |  |
| MI as cause  (n=815) | Circadian | Mesor | 4.17 ± 0.35 | <0.0001 | 4.17 ± 0.15 | | <0.0001 |  |
|  |  | Amplitude | 2.24 ± 0.50 | 0.0002 | 2.49 ± 0.21 | | <0.0001 |  |
|  |  | Acrophase | 14.09 ± 0.85 | <0.0001 | 14.33 ± 0.33 | | <0.0001 |  |
| 1:1 matched  (n=1,898) | Circadian | Mesor | 4.17 ± 0.20 | <0.0001 | 4.17 ± 0.14 | | <0.0001 |  |
|  |  | Amplitude | 1.35 ± 0.29 | 0.0001 | 1.34 ± 0.20 | | <0.0001 |  |
|  |  | Acrophase | 14.93 ± 0.81 | 0.0016 | 13.85 ± 0.56 | | 0.0036 |  |

Results are presented as the estimated coefficient ± SE.

P-values represent the significance of the individual parameters to the nonlinear regression model.

**eTable 2. Cosinor values of the OHCA occurrence shown by diabetes mellitus status in DANCAR.**

| Population | Time | Cosinor parameters | Diabetics | | Non-Diabetics | | |
| --- | --- | --- | --- | --- | --- | --- | --- |
|  |  |  | Coefficient ± SE | P-value | Coefficient ± SE | P-value |  |
| Total  (n=12,734) | Circadian | Mesor | 4.17 ± 0.19 | <0.0001 | 4.18 ± 0.17 | <0.0001 |  |
|  |  | Amplitude | 1.40 ± 0.27 | <0.0001 | 1.68 ± 0.23 | <0.0001 |  |
|  |  | Acrophase | 13.17 ± 0.74 | 0.13 | 13.48 ± 0.42 | 0.0113 |  |
|  | Septadian | Mesor | 14.43 ± 0.32 | <0.0001 | 14.14 ± 0.23 | <0.0001 |  |
|  |  | Amplitude | 0.47± 0.46 | 0.36 | 0.63 ± 0.33 | 0.13 |  |
|  |  | Acrophase | 7.37 ± 1.07 | 0.0005 | 7.22 ± 0.58 | 0.0002 |  |
| MI as cause  (n=791) | Circadian | Mesor | 4.17 ± 0.26 | <0.0001 | 4.17 ± 0.16 | <0.0001 |  |
|  |  | Amplitude | 1.71 ± 0.36 | 0.0001 | 1.87 ± 0.23 | <0.0001 |  |
|  |  | Acrophase | 12.79 ± 0.81 | 0.34 | 14.07 ± 0.46 | 0.0002 |  |
| 1:1 matched  (n=4,962) | Circadian | Mesor | 4.17 ± 0.19 | <0.0001 | 4.17 ± 0.20 | <0.0001 |  |
|  |  | Amplitude | 1.40 ± 0.27 | <0.0001 | 1.57 ± 0.28 | <0.0001 |  |
|  |  | Acrophase | 13.12 ± 0.74 | 0.14 | 13.31 ± 0.69 | 0.07 |  |

Results are presented as the estimated coefficient ± SE.

P-values represent the significance of the individual parameters to the nonlinear regression model.

**eTable 3. Double cosinor values of the OHCA occurrence shown by diabetes mellitus status in ARREST and DANCAR.**

| Population | Time | parameters | Diabetics | | | Non-Diabetics | | | |
| --- | --- | --- | --- | --- | --- | --- | --- | --- | --- |
|  |  |  | Coefficient ± SE | P-value | Coefficient ± SE | | P-value |  |  |
| Total | ARREST | Mesor | 4.17 ± 0.18 | <0.0001 | 4.17 ± 0.08 | | <0.0001 |  |  |
|  |  | Amplitude 1 | 1.36 ± 0.25 | <0.0001 | 1.79 ± 0.11 | | <0.0001 |  |  |
|  |  | Acrophase 1 | -1963108 ± 0.70 | <0.0001 | -535904 ± 0.23 | | <0.0001 |  |  |
|  |  | Amplitude 2 | 0.70 ± 0.25 | 0.0116 | 0.78 ± 0.11 | | <0.0001 |  |  |
|  |  | Acrophase 2 | 7294613 ± 0.68 | <0.0001 | 48246366 ± 0.27 | | <0.0001 |  |  |
|  |  | Peak 1, h | 11.8 | | 12.1 | | | |  |
|  |  | Peak 2, h | 19.9 | | NA | | |  |  |
|  | DANCAR | Mesor | 4.17 ± 0.12 | <0.0001 | 4.18 ± 0.001 | | <0.0001 |  |  |
|  |  | Amplitude 1 | 1.40 ± 0.17 | <0.0001 | 1.68 ± 0.002 | | <0.0001 |  |  |
|  |  | Acrophase 1 | 13.17 ± 0.46 | <0.0001 | 13.48 ± 0.34 | | <0.0001 |  |  |
|  |  | Amplitude 2 | 1.00 ± 0.17 | <0.0001 | -0.85± 0.002 | | <0.0001 |  |  |
|  |  | Acrophase 2 | 15777 ± 0.32 | <0.0001 | 15.93 ± 0.34 | | <0.0001 |  |  |
|  |  | Peak 1, h | 10.4 | | 11.1 | | |  |  |
|  |  | Peak 2, h | 20.1 | | 19.3 | | |  |  |
| MI as cause | ARREST | Mesor | 4.17 ± 0.34 | <0.0001 | 4.17 ± 0.14 | | <0.0001 |  |  |
|  |  | Amplitude 1 | 2.24 ± 0.48 | 0.0002 | 2.49 ± 0.20 | | <0.0001 |  |  |
|  |  | Acrophase 1 | 14.09 ± 0.82 | <0.0001 | 14.33 ± 0.30 | | <0.0001 |  |  |
|  |  | Amplitude 2 | 0.93 ± 0.48 | 0.069 | 0.46 ± 0.20 | | 0.0308 |  |  |
|  |  | Acrophase 2 | 27052470 ± 0.99 | <0.0001 | 1523924 ± 0.82 | | <0.0001 |  |  |
|  |  | Peak 1, h | 11.1 | | 13.4 | | |  |  |
|  |  | Peak 2, h | NA | | NA | | |  |  |
|  | DANCAR | Mesor | 4.17 ± 0.18 | <0.0001 | 4.17 ± 0.13 | | <0.0001 |  |  |
|  |  | Amplitude 1 | 1.71 ± 0.26 | <0.0001 | 1.87 ± 0.18 | | <0.0001 |  |  |
|  |  | Acrophase 1 | 12.79 ± 0.57 | <0.0001 | 14.07 ± 0.37 | | <0.0001 |  |  |
|  |  | Amplitude 2 | 1.25 ± 0.26 | 0.0001 | 0.67 ± 0.18 | | 0.0016 |  |  |
|  |  | Acrophase 2 | 6070.7 ± 0.39 | <0.0001 | -2641.6 ± 0.52 | | <0.0001 |  |  |
|  |  | Peak 1, h | 11.2 | | 12.0 | | |  |  |
|  |  | Peak 2, h | 21.7 | | NA | | |  |  |
| 1:1 matched | ARREST | Mesor | 4.17 ± 0.18 | <0.0001 | 4.17 ± 0.13 | | <0.0001 |  |  |
|  |  | Amplitude 1 | 1.36 ± 0.26 | <0.0001 | 1.34 ± 0.18 | | <0.0001 |  |  |
|  |  | Acrophase 1 | -977623 ± 0.72 | <0.0001 | 13.85 ± 0.52 | | <0.0001 |  |  |
|  |  | Amplitude 2 | 0.70 ± 0.26 | 0.014 | 0.44 ± 0.18 | | 0.024 |  |  |
|  |  | Acrophase 2 | 38475340 ± 0.70 | <0.0001 | 9642672 ± 0.78 | | <0.0001 |  |  |
|  |  | Peak 1, h | 11.8 | | 12.1 | | |  |  |
|  |  | Peak 2, h | 19.9 | | NA | | |  |  |
|  | DANCAR | A | 4.17 ± 0.12 | <0.0001 | 4.17 ± 0.14 | | <0.0001 |  |  |
|  |  | B | 1.40 ± 0.17 | <0.0001 | 1.57 ± 0.20 | | <0.0001 |  |  |
|  |  | C | 13.12 ± 0.46 | <0.0001 | 13.31 ± 0.48 | | <0.0001 |  |  |
|  |  | D | 1.01 ± 0.17 | <0.0001 | 0.97 ± 0.20 | | 0.0001 |  |  |
|  |  | E | 4989.4 ± 0.31 | <0.0001 | 1773.7 ± 0.39 | | <0.0001 |  |  |
|  |  | Peak 1, h | 10.4 | | 10.7 | | |  |  |
|  |  | Peak 2, h | 20.1 | | 19.9 | | |  |  |

Results are presented as the estimated coefficient ± SE.

P-values represent the significance of the individual parameters to the nonlinear regression model.
